# Application of extracorporeal shockwave therapy to improve microcirculation in diabetic foot ulcers: A prospective study

**DOI:** 10.1097/MD.0000000000033310

**Published:** 2023-03-17

**Authors:** Daun Jeong, Jin Hyuck Lee, Gyu Bin Lee, Ki Hun Shin, Jangsun Hwang, Se Youn Jang, Jin Yoo, Woo Young Jang

**Affiliations:** a Department of Orthopedic Surgery, Korea University College of Medicine, Seoul, Korea; b Institute of Nano, Regeneration, Reconstruction, Korea University, Seoul, Korea; c Department of Sports Medical Center, Korea University College of Medicine, Seoul, Korea; d Center for Biomaterials, Biomedical Research Institute, Korea Institute of Science and Technology (KIST), Seoul, Korea.

**Keywords:** diabetic foot ulcer, extracorporeal shockwave therapy (ESWT), ischemia, microcirculation, peripheral arterial disease (PAD)

## Abstract

Extracorporeal shockwave therapy (ESWT) can induce wound healing by increasing tissue microcirculation. However, studies on the effect of ESWT on enhancing tissue microcirculation in diabetic foot ulcer (DFU), particularly on when the microcirculation increases after ESWT application, are still lacking. Therefore, we aimed to examine the effectiveness of ESWT in promoting microcirculation in DFU patients in a time-dependent manner. We included 50 feet of 25 patients with type 2 diabetes mellitus and Wagner grade I to II DFU in this study. The affected feet were used as the ESWT group and the unaffected contralateral feet were used as the control group. ESWT was performed in 3 sessions per week for a total of 3 weeks. Transcutaneous partial oxygen pressure (TcPO_2_) was used to evaluate the tissue microcirculation. The TcPO_2_ level (>43 mm Hg) in the ESWT group was recovered by the 2nd week of treatment, and statistical significance (*P* < .05) was demonstrated at the same time. From the 2nd week of ESWT, a significant increase in TcPO_2_ was observed in Wagner grade I and II DFU. These findings imply that the ESWT may improve microcirculation in patients with Wagner grades I to II DFU. However, this impact requires at least 2 weeks or more than 6 sessions. For better comparison, further studies with larger clinical groups and extended period are needed.

## 1. Introduction

Diabetic foot ulcer (DFU) is a common complication in patients with diabetes mellitus (DM), and previous studies reported that the lifetime risk in patients with DM is approximately 15%.^[1]^ DFU is defined as foot ulcers in people with DM that are accompanied by neuropathy or peripheral artery disease (PAD).^[2]^ In particular, PAD is known as the major risk factor in patients with DM, and 10% to 15% of foot ulcers remain unhealed, while 5% to 24% of patients undergo lower-limb amputation, including foot or symes, within 6 to 18 months after the initial diagnosis of DFU.^[3–5]^ Diabetic foot complications are often caused by dysfunction of microcirculation, which is frequently accompanied by peripheral neuropathy and PAD.^[6]^ Considering the well-known importance of microcirculation in wound healing and recovery, it is essential to improve microcirculation in patients with DFU.^[7]^

Many techniques have been used to induce healing of DFU by increasing microcirculation in the ulcer area, such as hyperbaric oxygen therapy and negative pressure wound therapy, but there are limitations, such as pain and high cost.^[8,9]^ Extracorporeal shockwave therapy (ESWT) has recently been used to cope with these limitations.^[10,11]^ ESWT is a simple, inexpensive, and safe method that has been proven effective in various fields, such as the treatment of plantar fasciitis and lithotripsy.^[12–14]^ ESWT improves microcirculation and increases blood perfusion to the applied area.^[15,16]^ Hence, in patients with DFU, it is important to evaluate microcirculation after ESWT; transcutaneous partial oxygen pressure (TcPO_2_) has recently been used for this purpose.^[17,18]^ TcPO_2_ is noninvasive and can quantify the oxygenation of the skin; it can also be applied as an indicator of the improvement of ischemia due to surgical procedures or medications.^[19]^ Also, TcPO_2_ measurement is considered a reliable and suitable method for the follow-up of DFUs.^[20]^ However, despite the advantages of ESWT shown in previous studies,^[12–14,17]^ there is still a lack of studies on the effect of ESWT to improve microcirculation using TcPO_2_ in patients with DFU, especially when microcirculation increases after ESWT application.

Therefore, in this study, we aimed to examine the effectiveness of ESWT in promoting microcirculation in DFU patients in a time-dependent manner. We hypothesized that TcPO_2_ levels would change significantly after 3 weeks of ESWT, as described in previous studies.^[16,21]^

## 2. Methods

### 2.1. Study subjects

**Figure 1.**
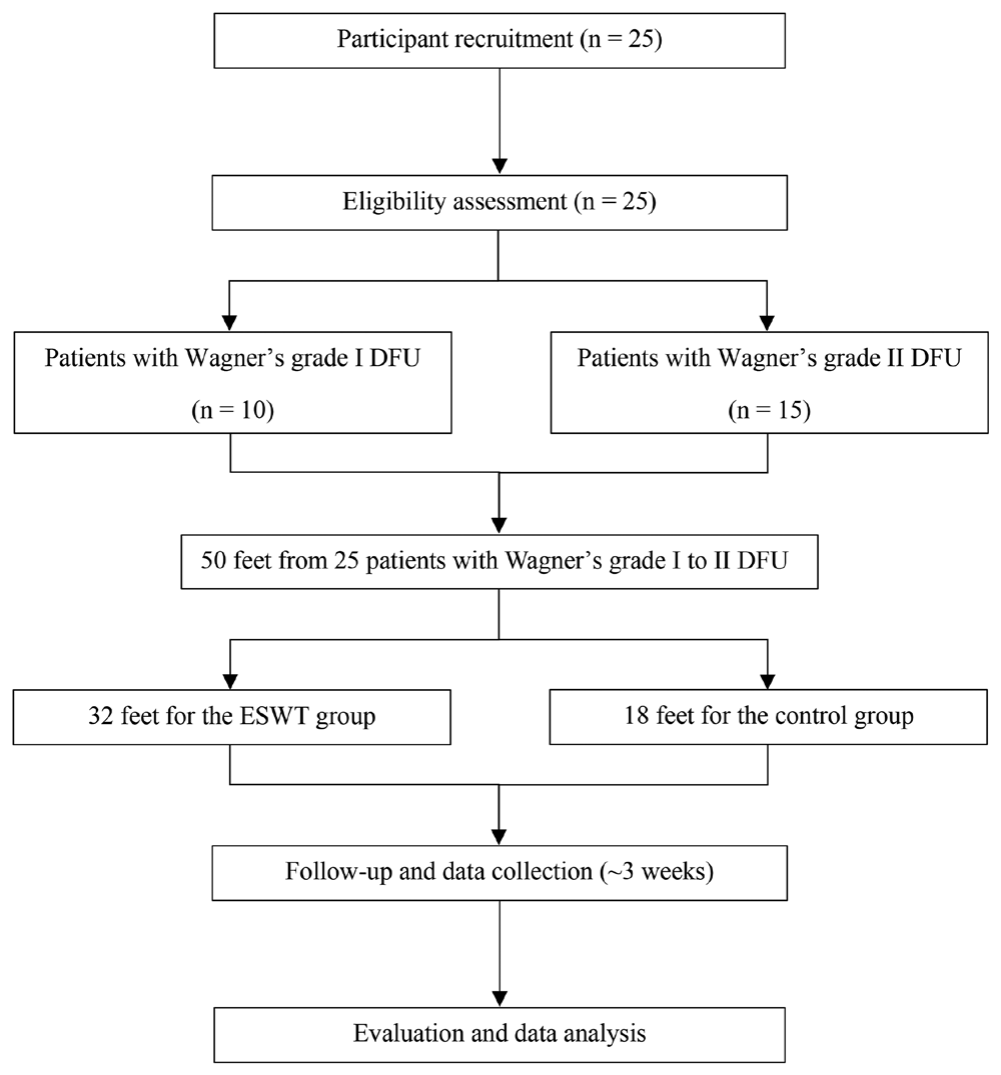
The patients consort diagram of the study.

This prospective study was approved by the institutional review board (IRB No. 2021AN0327) of our institution. The study was conducted in compliance with the principles of the Declaration of Helsinki and written informed consent was obtained from all patients before the study. 25 type 2 diabetes mellitus patients (19 males and 6 females) with DFU were recruited at our institution from July 2021 to May 2022. The inclusion and exclusion criteria are specified in Table 1, and only Wagner Classification^[22]^ grades I to II were included in this study; Wagner grade I is superficial ulcer, and Wagner grade II is deep ulcer extending into the tendon, ligament, or bone, but no abscess. Patients with an ulcer of <2.5 cm in diameter on foot were included,^[23,24]^ but patients with an ulcer area of lesser than 0.25 cm^2^ were excluded.^[21]^ The ulcerated feet comprised the ESWT group, while the contralateral feet without ulcers comprised the control group (50 feet from 25 patients).^[25]^ ESWT group: n = 32 (Wagner grade I, 14 feet; Wagner grade II, 18 feet) and control group (n = 18). The mean age of the participants was 67.8 ± 12.7 years (minimum age: 29 years and maximum age: 83 years). Figure 1 shows the patients consort diagram of the study.

**Table 1:** Inclusion and exclusion criteria of the study.

Inclusion criteria
● Patients aged 20 yr or older, male or female
● Patients with diabetes mellitus type 2 (T2DM) for 3 yr or more
● Patients with diabetic ulcers <2.5 cm in diameter on the foot (corresponding to Wagner grade I and II)
● Patients with less than 1 diabetic foot ulcer located on the surface of the foot and plantar surface 30 d before the first visit
● Patients who have understood and agreed in writing to all clinical trial requirements and treatment modalities
Exclusion criteria
● Women who are currently pregnant or lactating
● Patients with ulcer area <0.25 cm^2^
● Patients with known or suspected systemic infection
● Patients already enrolled in another clinical trial
● Patients who have received growth factor therapy (e.g., autologous platelet plasma, stem cells, becaplermin application, cell therapy, skin substitute, amnion tissue, extracellular matrix) within 30 d of the trial registration
● Patients who received radiation or chemotherapy within 3 mo of the trial enrollment
● Patients with suspected or progressing cellulitis at the target ulcer site at first or second visit
● Patients with osteomyelitis of the foot or ankle
● For patients with a history of osteomyelitis, systemic antibacterial treatment must have been completed 60 d before the start of the trial. Patients are excluded from this study if some are administered during the trial.
● Patients with diabetic neuroarthrosis
● Patients who have difficulty participating in the trial due to the investigator judgment or other reasons
● Patients who do not intend to participate in the trial

### 2.2. Production and application of extracorporeal shock waves (ESWT)

We used orthoPACE device (Sanuwave, Suwanee, GA) to deliver ESWT in the foot ulcer area. Before ESWT application, to prevent cross-contamination, the foot ulcer site was thoroughly cleaned and disinfected with medical sanitizer, and a sterile medical film was attached. To enable efficient energy transmission, the probe head was directly applied to the foot ulcer site using ultrasound gel. Each foot received 1500 impulses at an energy flux density of 0.2 mJ/mm^2^ with a frequency of 4 Hz.^[26]^ ESWT was applied 3 times a week, and each patient received 9 sessions over the course of 3 weeks. After each treatment, the therapist checked the wound area for adverse events, including infection, dermatitis, or necrosis. During the treatment, no systemic or neurovascular complications related to the device were observed. Based on the standard management guidelines for DFUs, all patients underwent adjunctive standard dressing care and debridement in combination with the ESWT application.^[27,28]^

### 2.3. TcPO_2_ measurement

TcPO_2_ (mm Hg) was measured using a TCM 400 device (Radiometer, Copenhagen, Denmark) to evaluate the microcirculation in the feet before and after ESWT. Sensors were attached to the skin of the dorsal part of the foot, in the first intermetatarsal space based on previous studies.^[29,30]^ The electrodes were warmed to 43ºC to 43.5ºC before the measurement to reduce the effect of temperature. The laboratory temperature was set at 25ºC to 26ºC^[16]^ owing to the oxygen permeability. All the measurements were performed at room temperature for 15 to 20 minutes. Baseline TcPO_2_ was measured before treatment, and follow-up measurements were conducted once a week during the total period. Based on a previous study,^[31]^ the optimal cutoff level of TcPO_2_ was 43 mm Hg, and the odds ratio was 4.4. Hence, in this study, the mean cutoff level of TcPO_2_ was set to 43 mm Hg.

### 2.4. Statistical analysis

Based on the report of a previous study on TcPO_2_ in patients with DFU,^[16]^ an improvement of >19.6% in TcPO_2_ between before and after ESWT was deemed clinically significant. A priori power analysis was conducted to determine the sample size (an α level of 0.05, power of 0.8). From the results of a pilot study involving 5 feet in the ESWT group, the effect size (Cohen d: 0.722) was calculated, and it was determined that 14 feet in the ESWT group were required to identify a clinically meaningful improvement > 19.6% in TcPO_2_ between before and after the ESWT group. The power of this study was 0.819. Data are expressed as mean ± standard error of the mean unless otherwise stated. The Q-Q plots and Shapiro–Wilk test were used to test all data group for normality, and normal distribution was shown in all groups. One-way analysis of variance was used to compare the significance within each group, followed by a paired *t* test as a post hoc test. The significance of the differences between subgroups was analyzed using an independent *t* test. *P* < .05 is considered significant between groups. All statistical analyses were performed according to the suggestion of a statistician, using the Statistical Package for Social Sciences, version 26 (Statistical Package for Social Sciences Inc., Chicago, IL).

## 3. Results

**Figure 2.**
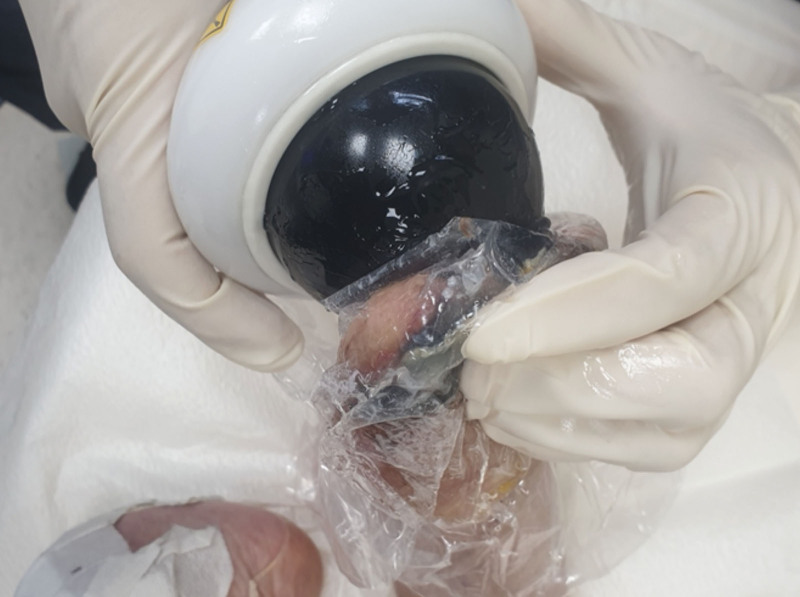
The photograph of ESWT on DFU. DFU = diabetic foot ulcer, ESWT = extracorporeal shockwave therapy.

**Figure 3.**
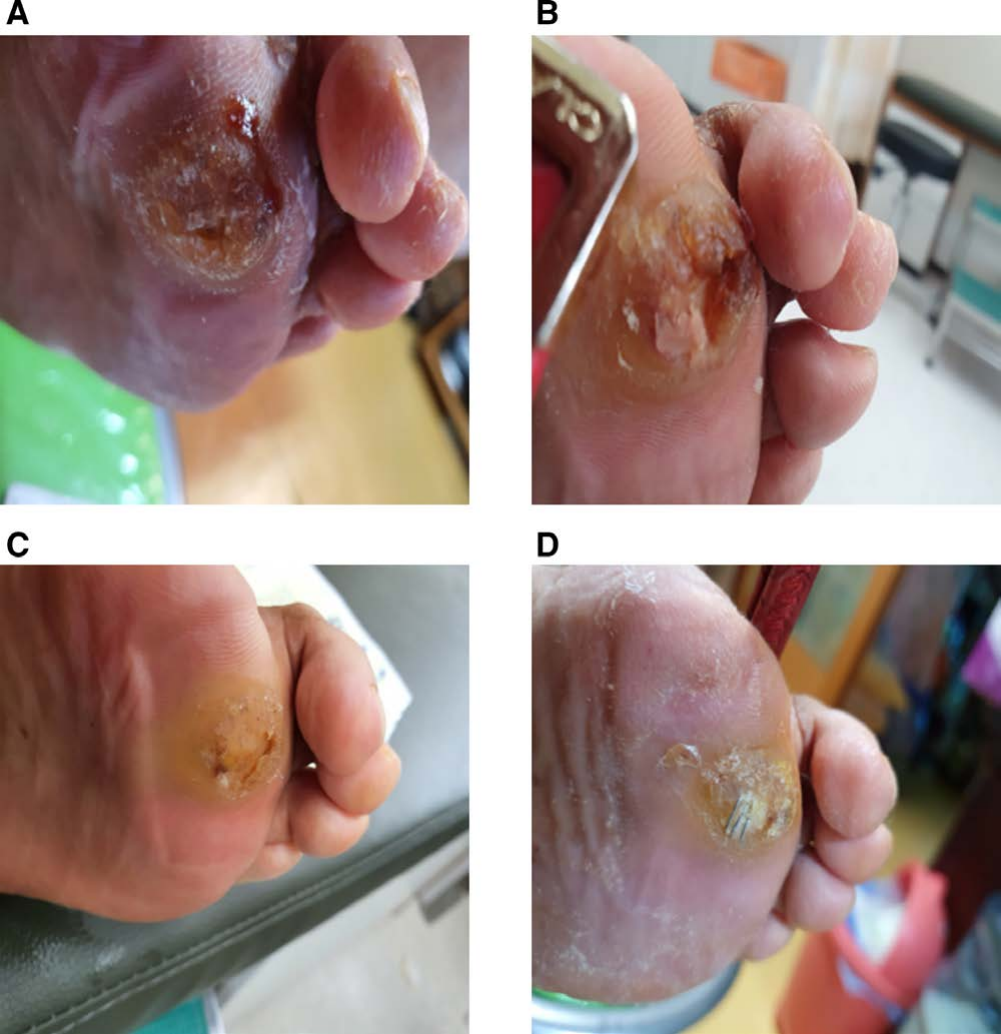
The representative photographs of the DFU before and after ESWT. (a) The patient first visit. (b) The 1st wk of ESWT. (c) The 2nd wk of ESWT. (d) The 3rd wk of ESWT. DFU = diabetic foot ulcer, ESWT = extracorporeal shockwave therapy.

The demographic characteristics of patients are presented in Table 2. The affected side of the feet was set as the treatment group (n = 32, 14 in Wagner grade I and 18 in Wagner grade II), and the unaffected feet were set as the control group (n = 18). A photograph of ESWT is shown in Figure 2. Figure 3 shows the representative images of DFU at each measurement time point.

**Table 2:** The demographic data of patients.

Variables	Total
Number of patients	25
Number of feet	50
Number of affected feet	32
Number of unaffected feet	18
Age (yr)	67.76 ± 12.7
Sex, n (%)	
Male	19 (76)
Female	6 (24)
Height (cm)	161.16 ± 8.95
Weight (kg)	60.80 ± 10.08
Body mass index (kg/m^2^)	23.36 ± 2.69
Wagner grade of DFUs, n (%)	
Wagner grade I	14 (43.75)
Wagner grade II	18 (56.25)

Data were expressed as mean ± standard deviation (SD).

DFU = diabetic foot ulcer.

### 3.1. Comparison of serial change of TcPO_2_ in each group

**Figure 4.**
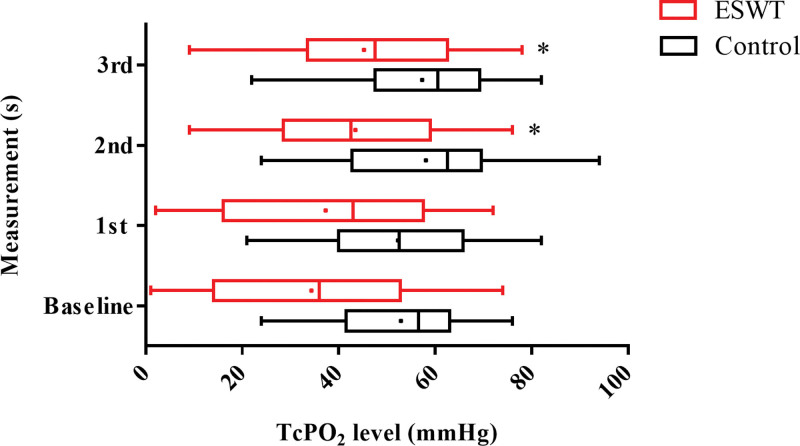
Serial change of TcPO_2_ of baseline and follow-up measurements. *P < .05 versus baseline (n = 32). Vertical line in each box represents the median TcPO_2_. Horizontal lines extending from each box represent the maximum and minimum TcPO_2_ for each group. The mean value for each group is indicated by a dot. ESWT = extracorporeal shockwave therapy, TcPO_2_ = transcutaneous partial oxygen pressure.

Figure 4 shows a comparison of serial changes in TcPO_2_ in each group. In the ESWT group (red lines), TcPO_2_ was significantly increased in 2nd and 3rd measurements compared to the baseline (baseline: 34.34 ± 3.92 mm Hg vs 1st measurement: 37.28 ± 3.93 mm Hg, *P* = .282, baseline vs 2nd measurement: 43.47 ± 3.47 mm Hg, *P* = .003, and baseline vs 3rd measurement: 45.25 ± 3.61 mm Hg, *P* = .001). In the control group (black lines), no significant differences (baseline: 52.89 ± 3.85 mm Hg vs 1st measurement: 52.33 ± 3.86 mm Hg, *P* = .806; baseline vs 2nd measurement: 58.06 ± 4.45 mm Hg, *P* = .209; and baseline vs 3rd measurement: 57.33 ± 3.42 mm Hg, *P* = .202) were found.

### 3.2. Comparison of the mean TcPO_2_ between the ESWT group and control group

**Figure 5.**
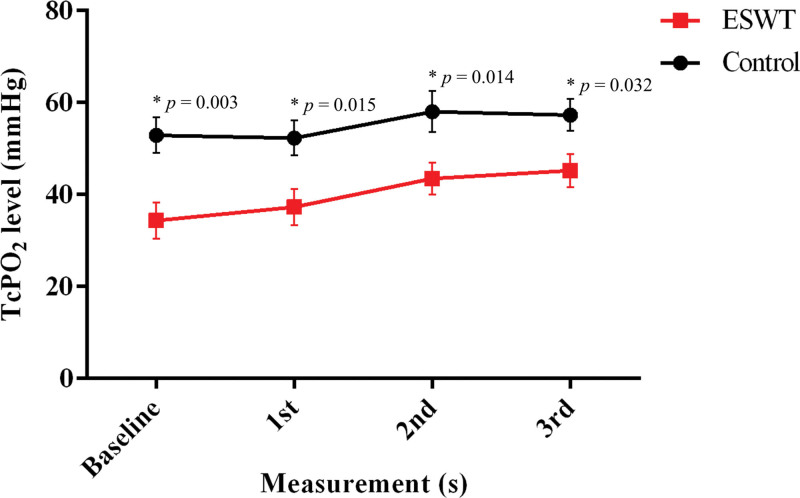
Comparison of TcPO_2_ between the ESWT groups and the control groups; *P < .05 indicates significance between the 2 groups (n = 32 for the ESWT group and n = 18 for the control group). ESWT = extracorporeal shockwave therapy, TcPO_2_ = transcutaneous partial oxygen pressure.

Figure 5 shows a comparison of the mean TcPO_2_ between the ESWT and control groups. The mean TcPO_2_ was significantly different between the groups (expressed as %p, ESWT group vs control group). The following were observed: baseline: 18.55 %p, *P* = .003; 1st measurement: 15.05 %p, *P* = .015; 2nd measurement: 14.59 %p, *P* = .014; and 3rd measurement: 12.08 %p, *P* = .032. A significant difference was found at all time points.

### 3.3. Comparison of TcPO_2_ in subgroups (Wagner grade I vs Wagner grade II)

**Figure 6.**
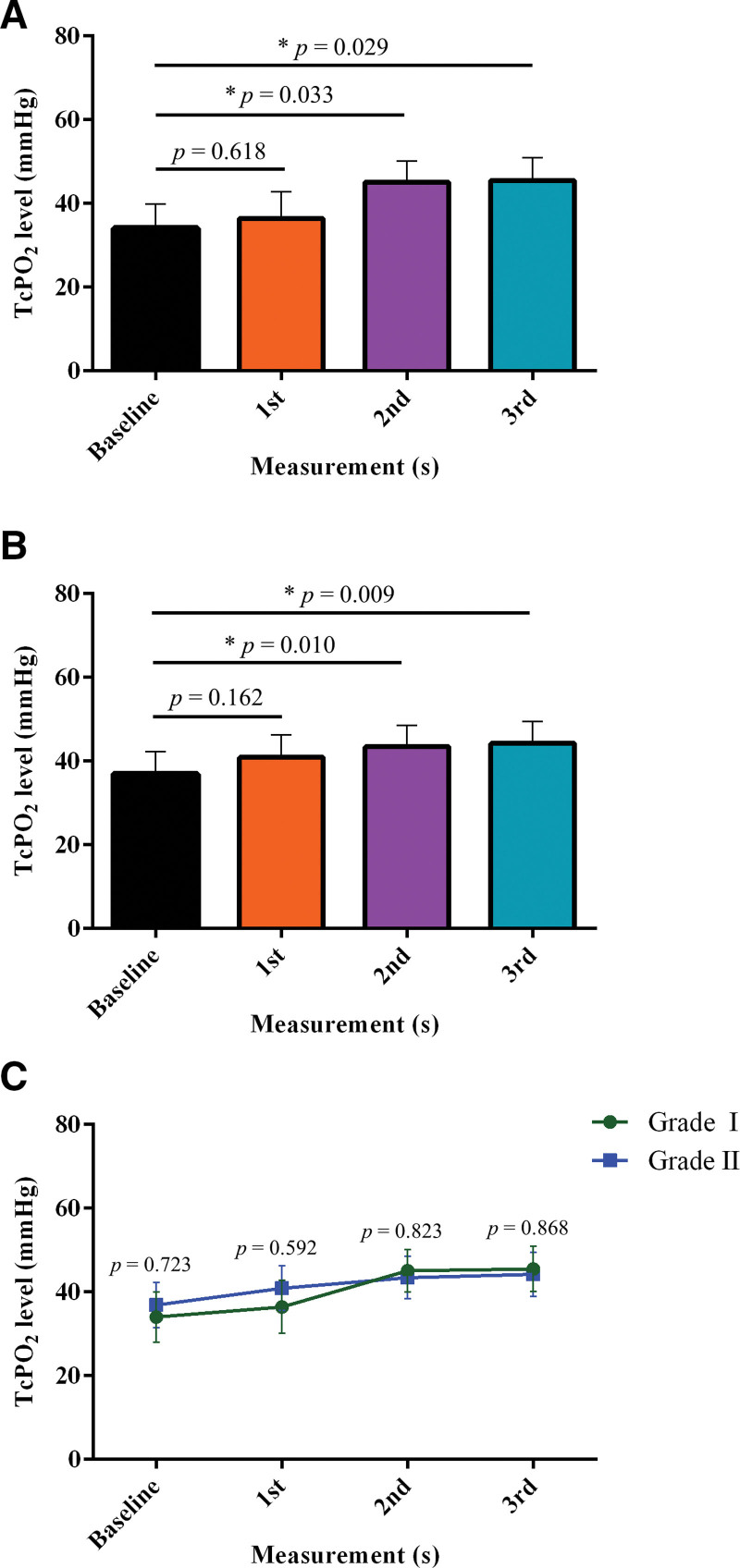
Comparison of TcPO_2_ between the subgroups. (a) Serial change of TcPO_2_ in the Wagner grade I; *P < .05 versus baseline (n = 14). (b) Serial change of TcPO_2_ in the Wagner grade II; *P < .05 versus baseline (n = 18). (c) Comparison of TcPO_2_ between the Wagner grade I and Wagner grade II. TcPO_2_ = transcutaneous partial oxygen pressure.

Figure 6 shows a comparison of serial changes in TcPO_2_ in the ESWT subgroup. For Wagner grades I and II, TcPO_2_ was significantly increased in 2nd and 3rd measurements compared to the baseline; for Wagner grade I (Fig. 6a), baseline: 34.00 ± 5.93 mm Hg vs 1st measurement: 36.43 ± 6.33 mm Hg, *P* = .618; baseline vs 2nd measurement: 45.07 ± 5.01 mm Hg, *P* = .033; and baseline vs 3rd measurement: 45.50 ± 5.41 mm Hg, *P* = .029. For Wagner grade II (Fig. 6b), baseline: 36.89 ± 5.43 mm Hg vs 1st measurement: 40.89 ± 5.33 mm Hg, *P* = .162; baseline vs 2nd measurement: 43.44 ± 5.03 mm Hg, *P* = .010; and baseline vs 3rd measurement: 44.22 ± 5.24 mm Hg, *P* = .009. Figure 6c shows no difference in the mean TcPO_2_ between the groups (expressed as %p, Wagner grade I—Wagner grade II. Baseline: −2.89 %p, *P* = .723, 1st measurement: −4.46 %p, *P* = .592, 2nd measurement: 1.63 %p, *P* = .823 and 3rd measurement: 1.28 %p, *P* = .868). No significant differences were found at any of the time points.

## 4. Discussion

The most important result of this study was that in the ESWT group, the mean TcPO_2_ level recovered to the optimal cutoff level (43 mm Hg in this study) at 2 weeks, and showed a statistical significance from 2nd week. However, the mean TcPO_2_ levels between the 2 groups were significantly different at all time points. Although this optimal cutoff level is still controversial, it is considered that a TcPO_2_ level above 40 mm Hg is sufficient for a good prognosis.^[16]^ Leenstra et al^[31]^ reported that a TcPO_2_ of 43 mm Hg or higher is associated with a good prognosis of DFU. In addition, Jang et al,^[32]^ reported that in the patient group with mild DFU, the healing rate of DFU was increased in patients with TcPO_2_ of 40 mm Hg or higher. Herein, the elevation of TcPO_2_ after ESWT above 43 mm Hg was observed as early as the 2nd week, indicating a good prognosis for DFU.

DFU is a complication of diabetes that can significantly increase morbidity and mortality.^[33]^ However, DFU can be prevented or delayed with early and appropriate medical intervention.^[34]^ The importance of blood flow and delivery of oxygen to the wound cannot be overemphasized for successful healing.^[16]^ Herein, 3 weeks of the ESWT treatment were performed on patients with Wagner grade I to II DFU, and changes in microcirculation in the foot were measured. We found restoration of TcPO_2_ with increased blood perfusion of the ulcers 2 weeks after ESWT in the patients with DFU compared to baseline conditions. This implies that 2 weeks after ESWT application, the expression of tissue regenerative factors that increase angiogenesis and vasodilation may be facilitated.^[35]^ Wound healing ability is impaired in patients with DFU.^[36]^ One of these factors is diabetic wound microcirculation dysfunction.^[6,37]^ In diabetic conditions, it is prone to becoming an ischemic condition due to a decrease in blood perfusion, and a high blood glucose concentration lowers the function of the inflammatory system, making it vulnerable to infection.^[38]^ In many patients with DFU, a decrease in vasodilators (i.e., nitric oxide) induces endothelial cell dysfunction, which in turn leads to decreased microcirculation.^[39]^ However, the application of ESWT is known to increase the activity of angiogenesis and tissue regeneration markers, such as von Willebrand factor, endothelial nitric oxide synthase, vascular endothelial growth factor, proliferating cell nuclear antigen, and epidermal growth factor.^[24,40,41]^ Therefore, the beneficial effect of ESWT increases microcirculation to the diabetic wound site and can also reduce ischemic conditions.^[42]^

Furthermore, in this study, although there was a difference in the mean TcPO_2_ levels in both baseline conditions at the 1st, 2nd, and 3rd measurements between the ESWT and control groups, the mean TcPO_2_ levels recovered to above 43 mm Hg (optimal cutoff level) at the 2nd and 3rd measurements with the application of ESWT. Given the results of this study, even if there is statistical significance in the difference in TcPO_2_ levels between the ESWT and control groups, careful interpretation is necessary. Yang et al^[16]^ reported that TcPO_2_ levels improved significantly after 3 weeks of ESWT application in 2 sessions per week in patients with DFU. Similarly, Jeppesen et al^[21]^ also found that TcPO_2_ levels increased after 3 weeks of ESWT application in 2 sessions per week in patients with DFU. However, in this study, the mean TcPO_2_ levels improved to optimal cutoff levels after 2 weeks because ESWT was applied for 3 sessions per week. This suggests that the application of ESWT for at least 2 weeks or more than 6 sessions may be required to improve blood flow at optimal cutoff levels in patients with DFU.

In particular, in this study, interesting results were found in the subgroup analysis; the mean TcPO_2_ levels were not different in both the baseline and the 1st, 2nd, and 3rd measurements in the affected feet between the Wagner grade I and II groups. In addition, in both Wagner grade I and II groups, restoration of the optimal cutoff range of TcPO_2_ with increased blood perfusion of the ulcers from 2 weeks after ESWT compared with the baseline. These findings indicate that DFU severity may not affect the effectiveness of ESWT. However, based on the results of this study, further studies are needed to determine whether the same results will be obtained for grade III or higher DFUs.

This study has some limitations. First, a quantitative comparison of wound healing would have been possible if the size (e.g., area and depth) of the ulcer before and after ESWT applications were compared. Furthermore, standard treatment criteria based on the depth or severity of the wound are required. Second, this study follow-up period was short. Previous systematic reviews,^[10,11]^ reported that the therapeutic effect of ESWT in improving DFU healing could be high; however, there was a deviation in the follow-up period. A high-quality study with long-term follow-up (i.e., a randomized controlled trial) may be needed to clarify the effect of ESWT in patients with DFU. Finally, more therapeutic parameters are required to investigate the therapeutic effect of ESWT in detail. Angiogenesis may be aided by wound healing markers, including vascular endothelial growth factor, platelet-derived growth factor, and fibroblast growth factor 1.^[43]^ Despite these limitations, there was little or no pain during the ESWT procedure; therefore, it may be less burdensome for the patient, both physically and economically. Furthermore, for therapists, the device can be easily managed and safely applied to patients in the clinical field. Hence, ESWT is a safe, effective treatment for both DFU patients and therapists.^[13,16,44,45]^

## 5. Conclusions

The results of the present study confirmed that ESWT significantly increased microcirculation in DFU and could be beneficial for the treatment of DFU. In addition, a period of ESWT application of at least 2 weeks or more than 6 sessions may be considered for the treatment of DFU with Wagner grades I and II. Therefore, since ESWT is a safe and effective method for treating DFU, it is worthy of active consideration as a standard treatment for DFU.

## Acknowledgments

The authors would like to appreciate Kove Inc. (Yangsan, Gyeongsangnam-do, Korea) for technical support of the orthoPACE device.

## Author contributions

**Conceptualization:** Daun Jeong, Jin Hyuck Lee, Woo Young Jang.

**Data curation:** Gyu Bin Lee, Ki Hun Shin.

**Formal analysis:** Daun Jeong, Jin Hyuck Lee.

**Funding acquisition:** Jangsun Hwang, Woo Young Jang.

**Investigation:** Jin Hyuck Lee, Gyu Bin Lee, Ki Hun Shin.

**Methodology:** Daun Jeong, Jin Hyuck Lee, Woo Young Jang.

**Project administration:** Jin Hyuck Lee, Woo Young Jang.

**Resources:** Jangsun Hwang, Se Youn Jang, Jin Yoo.

**Software:** Daun Jeong, Jin Hyuck Lee.

**Supervision:** Woo Young Jang.

**Validation:** Jin Hyuck Lee, Gyu Bin Lee, Ki Hun Shin.

**Visualization:** Daun Jeong, Jin Hyuck Lee.

**Writing – original draft:** Daun Jeong, Jin Hyuck Lee.

**Writing – review & editing:** Daun Jeong, Jin Hyuck Lee, Woo Young Jang.

## References

[1] Jeffcoate WJ Harding KG. Diabetic foot ulcers. Lancet. 2003;361:1545–51. 12737879 10.1016/S0140-6736(03)13169-8

[2] Alexiadou K Doupis J. Management of diabetic foot ulcers. Diabetes Ther. 2012;3:4. 22529027 10.1007/s13300-012-0004-9 PMC3508111

[3] Soyoye DO Abiodun OO Ikem RT. Diabetes and peripheral artery disease: a review. World J Diabetes. 2021;12:827–38. 34168731 10.4239/wjd.v12.i6.827 PMC8192257

[4] Katsilambros N Dounis E Makrilakis K. Atlas of the Diabetic Foot. 2nd ed. Chichester: John Wiley & Sons; 2010.

[5] Sun J-H Tsai J-S Huang C-H. Risk factors for lower extremity amputation in diabetic foot disease categorized by Wagner classification. Diabetes Res Clin Pract. 2012;95:358–63. 22115502 10.1016/j.diabres.2011.10.034

[6] Baltzis D Eleftheriadou I Veves A. Pathogenesis and treatment of impaired wound healing in diabetes mellitus: new insights. Adv Ther. 2014;31:817–36. 25069580 10.1007/s12325-014-0140-x

[7] Antonopoulos CN Lazaris A Venermo M. Predictors of wound healing following revascularization for chronic limb-threatening ischemia. Vasc Endovasc Surg. 2019;53:649–57. 10.1177/1538574419868863 31405350

[8] Meloni M Izzo V Vainieri E. Management of negative pressure wound therapy in the treatment of diabetic foot ulcers. World J Orthop. 2015;6:387–93. 25992316 10.5312/wjo.v6.i4.387 PMC4436907

[9] Lipsky BA Berendt AR. Hyperbaric oxygen therapy for diabetic foot wounds: has hope hurdled hype? Diabetes Care. 2010;33:1143–5. 20427686 10.2337/dc10-0393 PMC2858192

[10] Huang Q Yan P Xiong H. Extracorporeal shock wave therapy for treating foot ulcers in adults with type 1 and type 2 diabetes: a systematic review and meta-analysis of randomized controlled trials. Can J Diabetes. 2020;44:196–204.e3. 31515158 10.1016/j.jcjd.2019.05.006

[11] Hitchman LH Totty JP Raza A. Extracorporeal shockwave therapy for diabetic foot ulcers: a systematic review and meta-analysis. Ann Vasc Surg. 2019;56:330–9. 30496896 10.1016/j.avsg.2018.10.013

[12] Bhatia V Biyani CS. Vesical lithiasis: open surgery versus cystolithotripsy versus extracorporeal shock wave therapy. J Urol. 1994;151:660–2. 8308976 10.1016/s0022-5347(17)35041-3

[13] Mittermayr R Antonic V Hartinger J. Extracorporeal shock wave therapy (ESWT) for wound healing: technology, mechanisms, and clinical efficacy. Wound Repair Regen. 2012;20:456–65. 22642362 10.1111/j.1524-475X.2012.00796.x

[14] Dizon JNC Gonzalez-Suarez C Zamora MTG. Effectiveness of extracorporeal shock wave therapy in chronic plantar fasciitis: a meta-analysis. Am J Phys Med Rehabil. 2013;92:606–20. 23552334 10.1097/PHM.0b013e31828cd42b

[15] Tara S Miyamoto M Takagi G. Low-energy extracorporeal shock wave therapy improves microcirculation blood flow of ischemic limbs in patients with peripheral arterial disease: pilot study. J Nippon Med Sch. 2014;81:19–27. 24614391 10.1272/jnms.81.19

[16] Yang J-P Lee Y-N Son JW. The impact of extracorporeal shock wave therapy on microcirculation in diabetic feet: a pilot study. Adv Skin Wound Care. 2019;32:563–7. 31764146 10.1097/01.ASW.0000604180.54706.b2

[17] Padberg JFT Back TL Thompson PN. Transcutaneous oxygen (TcPO2) estimates probability of healing in the ischemic extremity. J Surg Res. 1996;60:365–9. 8598670 10.1006/jsre.1996.0059

[18] Zimny S Dessel F Ehren M. Early detection of microcirculatory impairment in diabetic patients with foot at risk. Diabetes Care. 2001;24:1810–4. 11574447 10.2337/diacare.24.10.1810

[19] Got I. Transcutaneous oxygen pressure (TcPO2): advantages and limitations. Diabetes Metab J. 1998;24:379–84. 9805653

[20] Fejfarova V Matusˇka J Jude E. Stimulation TcPO2 testing improves diagnosis of peripheral arterial disease in patients with diabetic foot. Front Endocrinol. 2021;12: 744195. 10.3389/fendo.2021.744195 PMC8704582 34956078

[21] Jeppesen S Yderstraede K Rasmussen B. Extracorporeal shockwave therapy in the treatment of chronic diabetic foot ulcers: a prospective randomised trial. J Wound Care. 2016;25:641–9. 27827284 10.12968/jowc.2016.25.11.641

[22] Shah P Inturi R Anne D. Wagner’s classification as a tool for treating diabetic foot ulcers: our observations at a suburban teaching hospital. Cureus. 2022;14:e21501. 35223277 10.7759/cureus.21501 PMC8861474

[23] Bashmakov YK Assaad-Khalil SH Abou Seif M. Resveratrol promotes foot ulcer size reduction in type 2 diabetes patients. Int Sch Res Notices. 2014;2014:816307. 10.1155/2014/816307 PMC3950537 24701359

[24] Yazdanpanah L Shahbazian H Nazari I. Prevalence and related risk factors of diabetic foot ulcer in Ahvaz, south west of Iran. Diabetes Metab Syndr: Clin Res Rev. 2018;12:519–24. 10.1016/j.dsx.2018.03.018 29602761

[25] Lavery LA Higgins KR Lanctot DR. Home monitoring of foot skin temperatures to prevent ulceration. Diabetes Care. 2004;27:2642–7. 15504999 10.2337/diacare.27.11.2642

[26] Jankovic D. Case study: shock waves treatment of diabetic gangrene. Int Wound J. 2011;8:206–9. 21385321 10.1111/j.1742-481X.2011.00779.x PMC7950628

[27] Everett E Mathioudakis N. Update on management of diabetic foot ulcers. Ann N Y Acad Sci. 2018;1411:153–65. 29377202 10.1111/nyas.13569 PMC5793889

[28] Niederauer MQ Michalek JE Liu Q. Continuous diffusion of oxygen improves diabetic foot ulcer healing when compared with a placebo control: a randomised, double-blind, multicentre study. J Wound Care. 2018;27(Sup9):S30–45. 30207844 10.12968/jowc.2018.27.Sup9.S30

[29] Yang C Weng H Chen L. Transcutaneous oxygen pressure measurement in diabetic foot ulcers: mean values and cut-point for wound healing. J Wound Ostomy Cont Nurs. 2013;40:585–9. 10.1097/WON.0b013e3182a9a7bf 24202221

[30] Rosfors S. Microcirculation at different parts of the foot in healthy subjects. J Non invasive Vasc Invest. 2016;1:005.

[31] Leenstra B de Kleijn R Kuppens G. Photo-optical transcutaneous oxygen tension measurement is of added value to predict diabetic foot ulcer healing: an observational study. J Clin Med. 2020;9:3291. 33066355 10.3390/jcm9103291 PMC7602180

[32] Jang S-Y Jeong T-W Han S-K. Transcutaneous oxygen pressure to predict wound healing in mild diabetic feet. Arch Plast Surg. 2011;38:585–9.

[33] Martins-Mendes D Monteiro-Soares M Boyko EJ. The independent contribution of diabetic foot ulcer on lower extremity amputation and mortality risk. J Diabetes Complicates. 2014;28:632–8. 10.1016/j.jdiacomp.2014.04.011 PMC4240944 24877985

[34] Rismayanti IDA Nursalam N Farida VN. Early detection to prevent foot ulceration among type 2 diabetes mellitus patient: a multi-intervention review. J Public Health Res. 2022;11:2752. 35315261 10.4081/jphr.2022.2752 PMC8973203

[35] Galkowska H Wojewodzka U Olszewski WL. Chemokines, cytokines, and growth factors in keratinocytes and dermal endothelial cells in the margin of chronic diabetic foot ulcers. Wound Repair Regen. 2006;14:558–65. 17014667 10.1111/j.1743-6109.2006.00155.x

[36] Liu Y Liu Y Deng J. Fibroblast growth factor in diabetic foot ulcer: progress and therapeutic prospects. Front Endocrinol (Lausanne). 2021;12:744868. 34721299 10.3389/fendo.2021.744868 PMC8551859

[37] Flynn M Tooke J. Aetiology of diabetic foot ulceration: a role for the microcirculation? Diabet Med. 1992;9:320–9. 1600701 10.1111/j.1464-5491.1992.tb01790.x

[38] Boulton AJ Cavanagh PR Rayman G. The Foot in Diabetes. 4th ed. Chichester: John Wiley & Sons; 2006:214–215.

[39] Babu AK Kumar MP Krupavaram B. Diabetic foot ulcer, antimicrobial remedies and emerging strategies for the treatment: an overview. Int J Health Sci. 2022;6:2835–50.

[40] Wang C-J Ko J-Y Kuo Y-R. Molecular changes in diabetic foot ulcers. Diabetes Res Clin Pract. 2011;94:105–10. 21742400 10.1016/j.diabres.2011.06.016

[41] Mittermayr R Hartinger J Antonic V. Extracorporeal shock wave therapy (ESWT) minimizes ischemic tissue necrosis irrespective of application time and promotes tissue revascularization by stimulating angiogenesis. Ann Surg. 2011;253:1024–32. 21372687 10.1097/SLA.0b013e3182121d6e

[42] Huemer G Meirer R Gurunluoglu R. 022 extracorporal shock wave is more effective than gene therapy with tgf-beta to reduce ischemic necrosis in a rat epigastric skin flap model. Wound Repair Regen. 2005;13:A4–A27. 10.1111/j.1067-1927.2005.130308.x 15953045

[43] Rai V Moellmer R Agrawal DK. Stem cells and angiogenesis: implications and limitations in enhancing chronic diabetic foot ulcer healing. Cells. 2022;11:2287. 35892584 10.3390/cells11152287 PMC9330772

[44] Wang C-J Wu R-W Yang Y-J. Treatment of diabetic foot ulcers: a comparative study of extracorporeal shockwave therapy and hyperbaric oxygen therapy. Diabetes Res Clin Pract. 2011;92:187–93. 21310502 10.1016/j.diabres.2011.01.019

[45] Mabrouk M Badr NM Taha MM. Effect of extracorporeal shock wave therapy on skin blood perfusion in patients with diabetic foot: randomized controlled trial. Int J Adv Res. 2015;3:689–96.

